# Assessment of Safety Culture at a Veterinary Teaching Hospital in the United States

**DOI:** 10.3389/fvets.2021.638764

**Published:** 2021-03-15

**Authors:** Lydia C. Love, Mari-Wells Hedgpeth, James B. Robertson, Steven L. Marks, Regina M. Schoenfeld-Tacher

**Affiliations:** ^1^Department of Molecular and Biomedical Sciences, College of Veterinary Medicine, NC State University, Raleigh, NC, United States; ^2^Department of Clinical Sciences, College of Veterinary Medicine, NC State University, Raleigh, NC, United States; ^3^Office of Research, College of Veterinary Medicine, NC State University, Raleigh, NC, United States

**Keywords:** veterinary safety culture, validation safety culture survey, veterinary teaching hospital, safety climate, patient safety

## Abstract

This study assessed the fidelity of an existing questionnaire regarding attitudes toward safety culture in an academic veterinary hospital setting and gathered baseline data on these attitudes in a local population. A cross-sectional study design was used to evaluate perceptions held by veterinary teaching hospital employees. An established veterinary safety culture survey was modified and administered as a confidential online survey to faculty, house officers, and professional staff of a veterinary teaching hospital in the United States. Confirmatory and exploratory factor analysis were conducted to compare the adapted survey to the established version. Descriptive statistics were used to characterize baseline safety culture. The adapted survey exhibited factor groupings that were mostly in agreement with, but slightly different from, the original instrument. In general, survey respondents outlined positive attitudes toward the various domains of safety culture, though we identified opportunities for improvement in some areas. An adapted veterinary safety culture survey can be applied to a veterinary teaching hospital in the United States to assess baseline data surrounding the culture of safety and to identify opportunities for focused improvement efforts.

## Introduction

Culture is the shared values that influence the attitudes and behaviors of individuals within an organization. The term “safety culture” was first used in reports detailing the systems failures that led to the 1986 nuclear disaster at Chernobyl, when it was suggested that high risk industries could reduce accidents and safety incidents through development of a positive safety culture (1). The idea that a systems approach to management of inevitable human error would be more effective than individualized attempts to perfect performance was rapidly adopted by the healthcare system. Although various interpretations of safety culture are used in the literature, the primary definition is “the product of individual and group beliefs, values, attitudes, perceptions, competencies, and patterns of behavior that determine the organization's commitment to quality and patient safety” (2). A more concise version is “those aspects of the organizational culture which will impact on attitudes and behavior related to increasing or decreasing risk” (3). Informally, safety culture can be described as “the way we do things around here” in relation to ensuring patient safety (4). Safety culture as a construct can be thought of as a “leading indicator” in the assessment of patient safety, as opposed to a “lagging indicator,” for example, morbidity and mortality (5). As such, assessment of safety culture can reveal potential issues in communication, teamwork, resources, and management strategies, delineating areas for targeted improvement efforts.

Several psychometrically validated instruments exist for assessment of safety culture in human healthcare organizations (6, 7). One group has developed an adaptation of the Safety Attitudes Questionnaire (SAQ), the Nottingham Veterinary Safety Culture Survey (NVSCS), for use in veterinary organizations in the United Kingdom (8) but no reports exist of the outcomes of deployment of this instrument. The purpose of this study was to explore the applicability of an adapted version of the NVSCS to a veterinary teaching hospital in the United States and characterize existing safety culture at that institution. Assuming that similarities in the practice of veterinary medicine are greater than the differences that can be attributed to local culture, we hypothesized that the survey would demonstrate similar psychometric properties when administered to a sample of veterinary professionals in a referral teaching hospital in the United States and that the four original factors identified—Factor 1 “Organizational safety systems and behaviors,” Factor 2 “Staff perceptions of management,” Factor 3 “Risk perceptions,” and Factor 4 “Teamwork and communication,” along with any latent constructs, would be reproducible across veterinary settings. In addition, we wanted to gather baseline information regarding attitudes reflective of patient safety culture at this institution and identify opportunities for intervention by analyzing survey responses.

## Materials and Methods

The NC State University (NCSU) Institutional Review Board (IRB) approved the research protocol (#19177). We formulated the NCSU Veterinary Safety Culture Survey (VSCS) as a closed confidential web-based survey conducted over the course of 1 month. It was open to the following self-identified employee groups with clinical responsibilities in the NC State Veterinary Hospital: faculty members/instructors, house officers (interns, residents, and fellows), and professional support staff (assistants and technicians). An email with a link to a secure web application^1^ was sent by an NCSU employee unassociated with the study to several centrally maintained electronic mailing lists (listservs) to announce the survey; and an email reminder was sent halfway through the data collection time period. In addition, announcements were made in various meetings by personnel unrelated to the study. Participants were able to take the survey from a personal or work computer. The survey was confidential for the purposes of definition by the IRB because demographic information, including professional role (faculty member, house officer, or technician), years as a veterinary professional, and years at NCSU College of Veterinary Medicine (CVM) (the last two indicated in 5-year increments), was collected that could potentially be triangulated to identify the respondent. However, the secure web application treated the survey as anonymous; no IP addresses were collected and no cookies were placed. Individual participants who could not complete the survey in one sitting were assigned a unique identification number to input upon return to the survey. It was not possible to prevent duplicate responses due to the anonymous treatment of respondents by the secure web-based application.

Informed consent was obtained prior to collection of any responses or demographic data. The electronically completed consent form notified the participant that data would be collected confidentially and for research purposes only. The purpose of the research, identity of the primary investigator, and projected time to complete the survey were outlined in the consent form, along with the risks to the individual of participation in the survey and potential benefits to the wider veterinary community. No incentives were offered for participation. A description of how the data would be stored, for how long, and how data might be shared was included. Data was stored in a central password-protected database on the application server.

The survey was developed by adapting the NVSCS (8) to an audience in the United States. Two statements derived from the SAQ but not included in the NVSCS were added to the revised NCSU survey: “I would feel safe having my own pet treated here” and “I believe errors are handled appropriately in this practice.” These statements were included in the present survey because the authors and pilot respondents felt that they could reveal important information about safety culture in a veterinary teaching hospital. In addition, one statement was substituted with a new item that more closely aligned with clinical norms at NCSU (when errors occur, a formalized investigation is conducted). Finally, the original statement concerning supervision of nurses and inexperienced veterinarians was split into three items, in order to evaluate perceptions of supervision of students, technicians and house officers, specifically in a teaching institution.

The resulting instrument encompassed 33 statements associated with a five-point Likert-scale (1 = strongly agree, 5 = strongly disagree), three demographic classification questions, and an open-ended question to collect narrative-based information about NCSU employee perceptions of safety culture. The instrument was piloted with three Diplomates of the American College of Veterinary Anesthesia and Analgesia, one Diplomate of the American College of Veterinary Cardiology, one Diplomate of the American College of Veterinary Surgery, one Diplomate of the American College of Veterinary Internal Medicine, and two Licensed Veterinary Technicians. Based on their feedback, we revised some wording and sentence structure. The final form of the survey was distributed over six digital pages.

Participants were able to review and change their responses prior to submission. No questions were mandatory and so partially complete surveys were accepted. Incomplete surveys were analyzed using pairwise deletion to avoid loss of responses.

Prior to statistical analysis, negatively worded items were recoded to score on the same scale as other items. For example, a response of strongly disagree was switched to a 5, while strongly agree was coded as a 1. Confirmatory factor analysis was used to determine if the prior factor groupings held true for this data set. Exploratory factor analysis was then conducted on the extended data to examine the factors for this data set. Cronbach's alpha was used to evaluate consistency within the pre-defined groups. Analyses were conducted in R version 3.6.2^2^ using the packages ltm^3^ and lavaan^4^

Two-way tables were generated comparing the demographic groups to responses to each of the non-demographic questions using the extension of the Fisher's exact test beyond 2 × 2 tables and any variable which was found to be significantly different from the null hypothesis of independence using a Bonferroni corrected cutoff was reported along with its observed distribution.

A Fisher's exact test revealed that there were no significant differences in response patterns across respondent demographic groups, except for two statements. Therefore, we pooled data among the remaining statements for statistical analysis. Statement responses were assessed for normality visually and with a Shapiro-Wilks test. Data were not normally distributed and median scores are reported.

## Results

Data was collected between January 6 and February 3, 2020 and 100 surveys were submitted. Of those, 80 surveys were completed, resulting in an 80% completion rate. Personnel participating included 38 faculty members/instructors, 18 house officers, and 45 staff members of 100, 105, and 150 possible participants, respectively. This resulted in response rates of 38, 17, and 30%. View rate and participation rate were not collected due to the anonymous treatment of the survey by the web-based application. Approximately 25% (21/80) of survey respondents provided narrative comments. Most participants selected 11–15 years as time spent as a veterinary professional and 1–5 years as time employed at NCSU CVM.

Confirmatory factor analysis (Table 1) was undertaken on the 31 safety statements shared by the NCSU VSCS and the NVSCS to compare data from our community with the original factors found. Demographic questions were not included. The a priori defined factors were ill-fitting of the data's structure (from chi-squared for model fit: *p* = 9.63 × 10^−29^, from testing RMSEA > 0.05: *p* < 0.001, CFI: 0.555, TLI: 0.517). The four a-priori defined factors were internally consistent in general except the third factor, corresponding to items about risk perception (Table 2).

**Table 1:** Loadings for confirmatory factor analysis, showing how the questions from the NVSCS performed at NCSU.

		**Confirmatory factor analysis loading**			
**Original Nottingham VSCS factor**	**Question text**	**Factor 1**	**Factor 2**	**Factor 3**	**Factor 4**
Organizational safety systems and behavior	I am given formal feedback on errors which happen in this practice	**0.535**	0.279	0.461	0.248
	Errors have led to positive change in this practice	0.229	0.424	**0.61**	−0.24
	When errors occur a formalized investigation is conducted	0.053	0.104	**0.897**	0.1
	The team discusses the results of error investigations	0.437	0.247	**0.576**	0.094
	There are procedures and systems in place to prevent errors happening in this practice	0.175	0.258	**0.616**	−0.019
	If I make an error my supervisor does not address it unless he/she is forced to	0.379	0.273	0.32	0.263
	I find it difficult to discuss errors in this practice	0.433	0.477	0.287	−0.08
	Errors are informally discussed amongst my team	0.026	−0.008	0.097	0.092
	House officers are adequately supervised and supported even at busy times	**0.614**	0.297	0.008	0.173
	Technicians are adequately supervised and supported even at busy times	**0.542**	**0.505**	0.166	0.073
	Students are adequately supervised and supported even at busy times	**0.52**	0.37	0.051	−0.035
Staff perceptions of management	I am scared of my supervisor	0.134	**0.775**	0.176	0.135
	I always feel able to question the decisions or actions of someone with more authority	0.306	**0.702**	0.043	−0.071
	If I make an error I worry that I will get into trouble with my supervisor	0.355	**0.641**	0.064	0.033
	I am sometimes intimidated by another member of my team	0.329	**0.549**	0.159	−0.045
	I feel that my supervisor supports me if I make an error	0.21	**0.699**	0.495	−0.043
	I respect my supervisor	0.425	**0.696**	0.342	−0.121
	I always speak up if I perceive a problem with patient safety during a procedure	−0.096	0.466	0.041	**0.511**
	The level of staffing in the practice is always sufficient to handle the number of patients	0.315	0.226	−0.008	0.347
Risk perceptions	When my workload becomes excessive my performance is impaired	−0.102	−0.18	0.202	**0.924**
	I am less effective at work when I am fatigued	0.2	−0.02	−0.113	**0.576**
	Patient safety is never compromised to get more work done	0.385	−0.168	−0.225	0.306
	Important information is often lost at shift change or patient transfer	**–0.696**	0.029	−0.347	−0.199
Teamwork and communication	At present there is good cooperation between veterinarians and technicians	**0.768**	0.293	0.313	−0.087
	People who work here treat each other with respect	**0.824**	0.275	0.268	−0.065
	Technician input is well-received in this practice	**0.775**	0.383	0.11	−0.154
	At present there is good cooperation between reception and clinical staff	0.419	0.139	0.279	0.144
	This is a good place to work	**0.697**	0.46	0.177	0.039
	I have the support from other personnel to care for my patients	**0.595**	**0.584**	0.262	0.012
	Communication breakdowns are common	**0.662**	0.177	0.079	−0.027
	It is easy for personnel here to ask questions if there is something they do not understand	**0.551**	**0.557**	0.071	0.23

*Demographic information was excluded from this analysis. Bold values indicate the factor(s) upon which the statement loads*.

**Table 2:** Cronbach alpha for confirmatory factor analysis of common questions from NCSU VSCS and NVSCS.

**Factor**	**1 Organizational safety systems and behavior**	**2 Staff perceptions of management**	**3 Risk perceptions**	**4 Teamwork and communication**
**Alpha**	**0.872**	**0.862**	**0.298**	**0.922**

An exploratory factor analysis was conducted to elucidate the underlying structure of potentially interrelated measures without imposing a pre-determined outcome. The found factors' proportion of variance explained were 0.262, 0.137, 0.111, and 0.069, respectively. Most factors largely contained clusters from one or two prior factors. The first factor, “Visible patient safety indicators,” contained most of the NVSCS questions labeled as “Teamwork and communication,” as well as elements from other factors regarding adequate supervision of trainees (Organizational safety systems and behaviors), and loss of information at shift changes (Risk perceptions). What is most striking to note is the high loading of the statement regarding “I would feel safe having my own pet treated here,” indicating that these factors might encompass a snapshot of readily visible patient safety behaviors. Factor 2, Staff perceptions of management, was composed entirely of items from the analogous factor (Staff perceptions of management) on the NVSCS and the additional SAQ item regarding how errors are handled in the practice. Factor 3, Error response, consisted solely of five items from the original organizational safety systems of the NVSCS (8). Finally, Factor 4, Stress recognition, was relatively sparse. It contained three of the five items pertaining to risk perception as identified in the NVSCS. The full factor loadings are provided in Table 3. Note that responses to demographic questions were not included in the factor analysis. The four factors in our exploratory analysis were internally consistent in general with the exception of the fourth factor, corresponding to stress recognition, which had a Cronbach alpha value of 0.63 (Table 4).

**Table 3:** Loadings for exploratory factor analysis, showing relationships among all the NCSU VSCS items.

		**Exploratory factor analysis loading**			
**Original factor**	**Question text**	**Factor 1 visible indicators of patient safety**	**Factor 2 perceptions of management**	**Factor 3 error response**	**Factor 4 stress recognition**
Organizational safety systems and behavior	I am given formal feedback on errors which happen in this practice	0.476	0.238	**0.568**	0.317
	Errors have led to positive change in this practice	0.349	0.321	**0.608**	−0.326
	When errors occur a formalized investigation is conducted	0.043	0.215	**0.812**	−0.045
	The team discusses the results of error investigations	0.399	0.266	**0.622**	0.164
	There are procedures and systems in place to prevent errors happening in this practice	0.238	0.222	**0.575**	−0.139
	If I make an error my supervisor does not address it unless he/she is forced to	0.271	0.403	0.419	0.463
	I find it difficult to discuss errors in this practice	0.456	0.492	0.307	0.102
	Errors are informally discussed amongst my team	0.02	−0.071	0.13	0.006
	House officers are adequately supervised and supported even at	**0.608**	0.27	−0.012	0.246
	Technicians are adequately supervised and supported even at bus	**0.616**	0.406	0.182	0.074
	Students are adequately supervised and supported even at busy times	**0.574**	0.316	0.018	0.072
Staff perceptions of management	I am scared of my supervisor	0.276	**0.807**	0.102	0.028
	I always feel able to question the decisions or actions of someone with more authority	0.445	**0.592**	0.07	−0.058
	If I make an error I worry that I will get into trouble with my supervisor	0.455	**0.615**	0.061	0.043
	I am sometimes intimidated by another member of my team	0.436	0.441	0.082	−0.035
	I feel that my supervisor supports me if I make an error	0.381	**0.652**	0.382	−0.158
	I respect my supervisor	0.585	**0.592**	0.271	−0.167
	I always speak up if I perceive a problem with patient safety during a procedure	−0.02	0.432	0.037	0.181
	The level of staffing in the practice is always sufficient to handle the number of patients	0.236	0.195	0.068	0.423
Risk perceptions	When my workload becomes excessive my performance is impaired	−0.265	−0.031	0.217	**0.598**
	I am less effective at work when I am fatigued	0.068	0.037	−0.094	**0.619**
	Patient safety is never compromised to get more work done	0.194	−0.098	−0.188	**0.657**
	Important information is often lost at shift change or patient transfer	–**0.584**	0.072	−0.426	−0.295
Teamwork and communication	At present there is good cooperation between veterinarians and technicians	0.407	0.075	0.258	0.107
	People who work here treat each other with respect	**0.748**	0.276	0.193	0.16
	Technician input is well-received in this practice	**0.745**	0.391	0.274	−0.11
	At present there is good cooperation between reception and clinical staff	**0.595**	0.185	0.149	0.31
	This is a good place to work	**0.644**	0.383	0.1	0.149
	I have the support from other personnel to care for my patients	0.407	0.075	0.258	0.107
	Communication breakdowns are common	**0.748**	0.276	0.193	0.16
	It is easy for personnel here to ask questions if there is something they do not understand	**0.745**	0.391	0.274	−0.11
Questions from SAQ only	I believe errors are handled appropriately in this practice	0.485	**0.523**	0.496	0.11
	I would feel safe having my own pet treated here	**0.767**	0.363	0.288	−0.049

*Bold values indicate the factor(s) upon which the statement loads*.

**Table 4:** Cronbach alpha for exploratory factor analysis of NCSU VSCS.

**Factor**	**1 Observable Patient Safety Indicators**	**2 Perceptions of Management**	**3 Error Response**	**4 Stress Recognition**
**Alpha**	**0.893**	**0.906**	**0.827**	**0.629**

### Assessment of NCSU CVM Safety Culture

Within the domain of “Visible patient safety indicators,” the majority (>50%) of respondents agreed or strongly agreed with the following statements: “At present, there is good cooperation between veterinarians and technicians;” “People who work here treat each other with respect;” “Technician input is well-received at this practice;” “I have the support from other personnel to care for my patients to the best of my ability;” “It is easy for personnel here to ask questions if there is something that they do not understand;” “I would feel safe having my own pet treated here;” and “This is a good place to work” (Table 5). On the other hand, the majority of respondents agreed or strongly agreed with the statement “Communication breakdowns are common” and almost half (46%; 36/78) agreed or strongly agreed that “Important information is often lost at shift change or patient transfer.”

**Table 5:** Responses to NCSU VSCS. Counts and percentages represent the text selected by participants.

	Median^*^	**Range**	**Std. Dev**.	**Strongly disagree (%)**	**Disagree (%)**	**Neutral (%)**	**Agree (%)**	**Strongly agree (%)**
I am given formal feedback on errors which happen in this practice (*n* = 73)	4	1–5	1.01	2.7	23.3	21.9	43.8	8.2
Errors have led to positive change in this practice (*n* = 73)	4	1–5	0.84	1.4	8.2	12.3	63.0	15.1
When errors occur, a formalized investigation is conducted (*n* = 68)	3	1–5	0.93	1.5	16.2	33.8	38.2	10.3
The team discusses the results of error investigations (*n* = 68)	3	1–5	1.06	5.9	26.5	22.1	39.7	5.9
There are procedures and systems in place to prevent errors happening in this practice (*n* = 77)	4	2–5	0.76	0	5.2	11.7	58.4	24.7
If I make an error my supervisor does not address it unless he/she is forced to^*^ (*n* = 69)	4	1–5	1.08	18.8	39.1	18.8	21.7	1.4
I find it difficult to discuss errors in this practice^*^ (*n* = 77)	4	1–5	1.19	18.2	41.6	16.9	15.6	7.8
Errors are informally discussed amongst my team (*n* = 77)	4	1–5	0.92	1.3	9.1	9.1	53.2	27.3
House officers are adequately supervised and supported, even at busy times (*n* = 70)	3	1–5	1.26	8.6	31.4	18.6	24.3	17.1
Technicians are adequately supervised and supported, even at busy times (*n* = 74)	3	1–5	1.16	9.5	27.0	20.3	35.1	8.1
Students are adequately supervised and supported, even at busy times (*n* = 73)	3	1–5	1.20	8.2	19.2	23.3	32.9	16.4
I am scared of my supervisor^*^ (*n* = 77)	5	2–5	0.97	53.2	27.3	10.4	9.1	0
I always feel able to question the decisions or actions of someone with more authority (*n* = 77)	3	1–5	1.21	14.3	27.3	23.4	26.0	9.1
If I make an error, I worry that I will get into trouble with my supervisor^*^ (*n* = 77)	4	1–5	1.21	19.5	33.8	18.2	22.1	6.5
I am sometimes intimidated by another member of my team^*^ (*n* = 77)	4	1–5	1.21	19.5	40.3	10.4	24.7	5.2
I feel that my supervisor supports me if I make an error (*n* = 74)	4	1–5	1.00	4.1	6.8	16.2	50.0	23.0
I respect my supervisor (*n* = 78)	5	1–5	1.14	3.8	7.7	11.5	21.8	55.1
I always speak up if I perceive a problem with patient safety during a procedure (*n* = 74)	4	2–5	0.75	0	4.1	8.1	52.7	35.1
The level of staffing in the practice is always sufficient to handle the number of patients (*n* = 77)	2	1–5	1.12	28.6	37.7	15.6	15.6	2.6
When my workload becomes excessive, my performance is impaired^*^ (*n* = 78)	2	1–5	0.95	3.8	6.4	10.3	59.0	20.5
I am less effective at work when I am fatigued^*^ (*n* = 78)	2	1–5	0.62	0	1.3	6.4	61.5	30.8
Patient safety is never compromised to get more work done (*n* = 75)	3	1–5	1.11	5.3	25.3	20.0	38.7	10.7
Important information is often lost at shift change or patient transfer (*n* = 63)	4	1–5	0.98	1.6	19.0	22.2	46.0	11.1
At present there is good cooperation between veterinarians and technicians (*n* = 75)	4	1–5	0.92	1.3	9.3	16.0	52.0	21.3
People who work here treat each other with respect (*n* = 78)	4	1–5	0.96	3.8	9.0	25.6	48.7	12.8
Technician input is well-received in this practice (*n* = 76)	4	1–5	1.05	5.3	7.9	18.4	47.4	21.1
At present, there is good cooperation between reception and clinical staff (*n* = 65)	4	2–5	0.83	0	10.8	23.1	53.8	12.3
This is a good place to work (*n* = 78)	4	1–5	0.92	2.6	2.6	17.9	44.9	32.1
I have the support from other personnel to care for my patients to the best of my ability (*n* = 73)	4	1–5	0.92	1.4	6.8	12.3	47.9	31.5
Communication breakdowns are common (*n* = 78)	2	1–5	1.13	19.2	37.2	19.2	20.5	3.8
It is easy for personnel here to ask questions if there is something that they do not understand (*n* = 78)	4	1–5	1.03	3.8	12.8	15.4	51.3	16.7
I believe errors are handled appropriately in this practice (*n* = 76)	4	1–5	0.92	1.3	14.5	23.7	50.0	10.5
I would feel safe having my own pet treated here (*n* = 76)	4	2–5	1.01	0	14.5	9.2	42.1	34.2

* *indicates reverse scored question*.

*Initial Likert scale 5 = strongly agree. Negatively worded questions were recoded to score on the same scale as other questions (strongly agree = 1) for purposes of calculating medians and factor analysis*.

Within the second factor, “Staff perceptions of management,” the majority of respondents agreed or strongly agreed with the following statements: “I respect my supervisor;” “I feel that my supervisor supports me if I make an error;” and “I believe errors are handled appropriately at this practice.” In addition, respondents disagreed or strongly disagreed with the statements “I am scared of my supervisor” and “If I make an error, I worry that I will get into trouble with my supervisor.” Unfortunately, the majority of respondents disagreed or strongly disagreed with the statement: “I always feel able to question the decision or actions of someone with more authority.”

Within the third domain, “Error response,” the majority of participants agreed or strongly agreed with the following two statements: “There are procedures and systems in place to prevent errors in this practice” and “Errors have led to positive change in this practice.” Responses to other items in this domain pertaining to formal management of error were distributed amongst the possible responses, though <50% (38/80) of respondents agreed or strongly agreed that: “I am given formal feedback on errors which happen in this practice.”

Within the fourth factor, “Stress recognition,” the majority of respondents agreed or strongly agreed with the following: “I am less effective at work when I am fatigued” and “When my workload becomes excessive, my performance is impaired.” Unfortunately, the majority of respondents were neutral, disagreed, or strongly disagreed with the statement that “Patient safety is never compromised to get more work done.”

Several statements did not load on any of the identified factors (Table 4). Responses to most of these items were positive regarding NCSU safety culture. Most respondents disagreed or strongly disagreed with the following: “If I make an error my supervisor does not address it unless forced to;” “I find it difficult to discuss errors in this practice;” and “I am sometimes intimidated by another member of my team” (Table 5). In addition, most respondents agreed or strongly agreed that “Errors are informally discussed amongst my team;” “I always speak up if I perceive a problem with patient safety during a procedure” and that “At present, there is good cooperation between veterinarians and technicians.” However, almost two-thirds (51/78) of survey respondents disagreed with the statement that “The level of staffing in the practice is always sufficient to handle the number of patients.”

**Figure 1:**
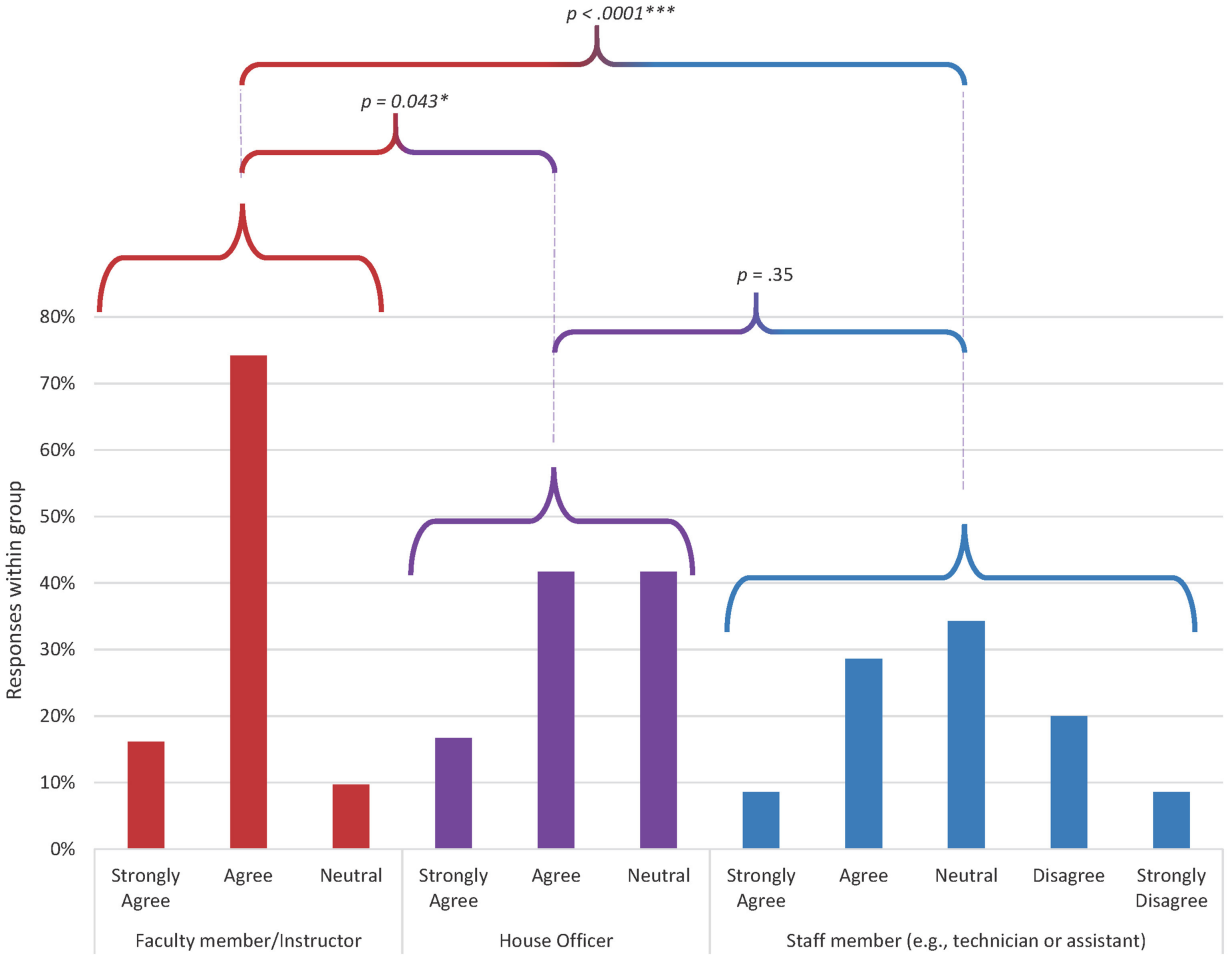
Distribution of responses by demographic group to the question “people who work here treat each other with respect” (*p* = 0.0005). Data from 31 faculty members, 12 house officers, and 35 staff members. Responses from faculty were significantly different from staff (*p* = 0.00009) and house officers (*p* = 0.043). There was no significant difference in response distribution between house officers and staff.

**Figure 2:**
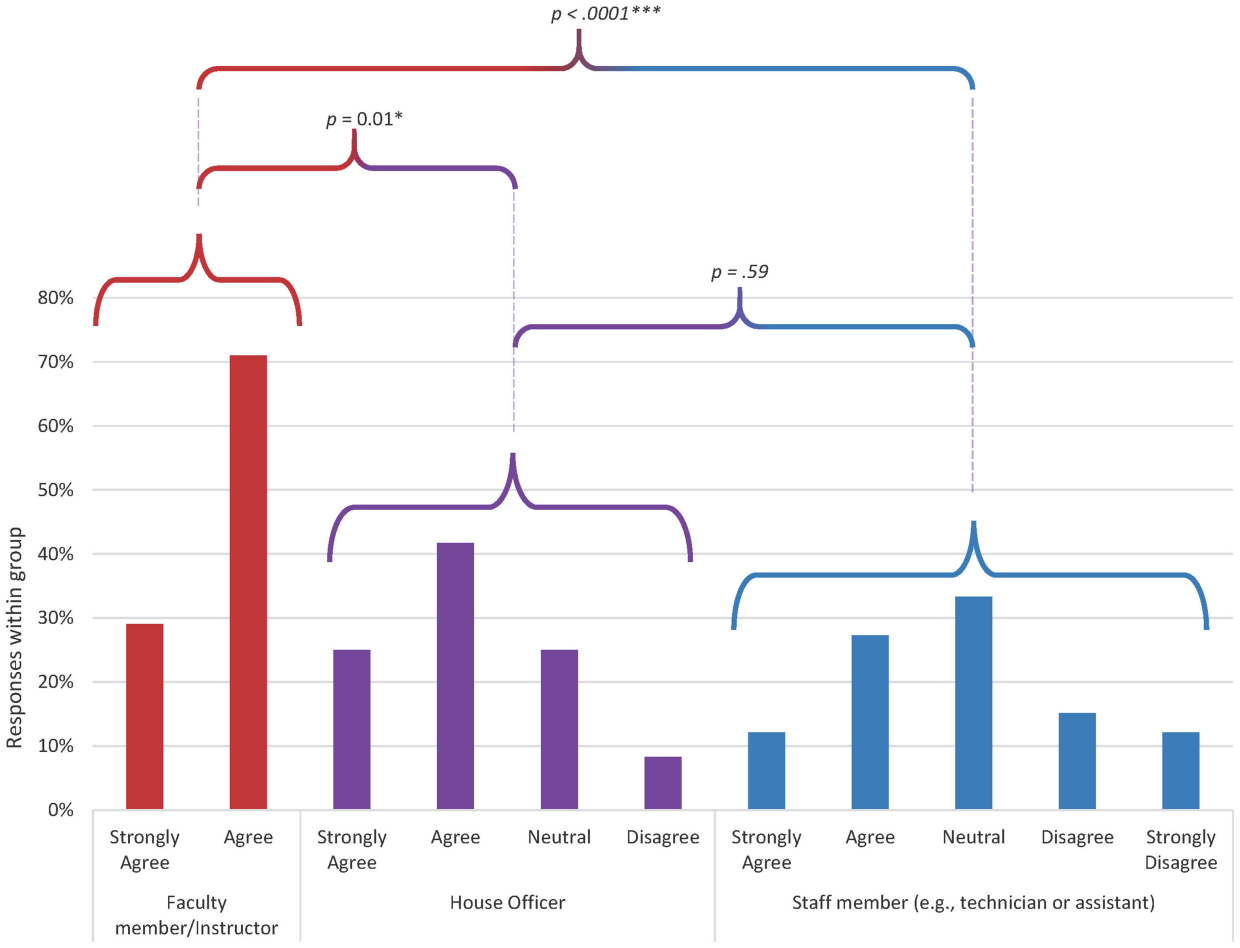
Distribution of responses by demographic group to the question “technician input is well-received in this practice” (*p* = 0.00003). Data from 31 faculty members, 12 house officers, and 33 staff members. Responses from faculty were significantly different from staff (*p* = 0.000001) and house officers (*p* = 0.01). There was no significant difference in response distribution between house officers and staff.

Only two statements demonstrated any pattern of difference amongst demographic groups (Figures 1, 2): “People who work here treat each other with respect” (*p* < 0.000) and “Technician input is well-received in this practice” (*p* < 0.000). In both cases, faculty members were more likely to agree with the sentiment than house officers or staff members.

There were 21 survey participants (6 faculty, 2 house officers, and 13 staff members) who submitted a response to the open-ended request for specific perspectives regarding patient safety and errors. Two of the authors (LCL and RMS-T) independently coded these responses, then met to discuss major themes and check for consistency. Each response was coded into one category. Three major themes were identified in the narrative statements: communication, the way in which errors are addressed, and the degree of variability at the service level. The importance of clear communication, and consequences of lapses in communication across departments were mentioned in 7 of the 21 responses. Concerns regarding how errors are addressed ranged from perceived favoritism to fear of repercussions and were mentioned in six of the comments. Variability in safety procedures and error avoidance techniques across services was noted in four responses. Other issues that were mentioned by one or two participants each included concerns about handling aggressive animals, perceived lack of support by supervisors, and a feeling of burnout. Faculty primarily commented on variability across services, while staff members were most concerned about issues that directly affected their own working conditions, such as under-staffing, inconsistent application of established safety protocols, and burnout.

## Discussion

Confirmatory and exploratory factor analysis of the NCSU VSCS revealed four domains that were internally consistent, but somewhat different to those identified by the NVSCS (8). In particular, items related to communication regrouped into new factors, indicating they were perceived differently by our audience compared with that of the NVSCS. Though it was possible that the similarities of veterinary practice in any setting could result in identification of similar latent constructs of safety culture, the differences in factor loading between the two surveys may be reflective of the fact that culture is created by shared experience and is therefore local in nature. Indeed, although certain values, beliefs, and resulting behaviors may be common among medical professionals, discrete organizational variances—even amongst clinical units in the same institution—appear to influence the construct of safety culture heavily (9, 10). Our results underscore the importance of validating safety culture survey instruments for the specific professional population of interest (11).

In the NCSU VSCS, our first factor was “Visible indicators of patient safety” and it aligned closely with Factor 4 (Teamwork and communication) from the NVSCS but also included three statements regarding supervision of trainees originally grouped in Factor 2 (“Organizational safety systems and behaviors”). With regards to the SAQ, items in this factor mostly included statements from the SAQ teamwork climate factor, but also from working conditions, perception of management, and, most importantly, safety climate (e.g., I would feel safe having my pet treated here). This reorganization of statements may reflect easily discernible indicators of patient safety; that is, these items may group together because they are institutional habits that employees in any position can readily perceive.

The second factor in the NCSU VSCS, “Perceptions of management” was reasonably aligned with the NVSCS Factor 2 (“Staff perceptions of management”) and encompasses statements that involve evaluation of the managerial behaviors that shape safety culture. The third factor, “Organizational safety systems” comprised five items from the third factor of the NVSCS, focusing exclusively on systems in place to investigate and mitigate errors. The fourth factor, “Stress recognition” corresponded to three items in this category on the NVSCS, and appear to all relate to the working conditions domain of the SAQ.

Regarding baseline safety culture assessment at NCSU, reported perceptions were generally encouraging. Responses within the first factor that organized around visible indicators of patient safety were particularly positive and this may reflect the easily identifiable behaviors related to the provision of safe patient care within the NC State Veterinary Hospital environments. However, the responses to a few of the statements within this domain delineated opportunity for improvement, especially regarding communication, as shown in Table 5. A little over half of respondents indicated that “Communication breakdowns are common” and almost half confirmed that “Important information is often lost at shift change or patient transfer.” In addition, several of the narrative statements coalesced around the issue of communication amongst team members, including: “When we have issues/errors with other services, it always tends to point to a lack of communication.,” “in some services … there is a gap of communication between faculty/residents/technicians and receptionists. There should be more group communication in the hospital as a whole.,” and that errors occur “.with patients being transferred to other departments without relevant history being disclosed.” Due to the multidisciplinary nature of advanced veterinary healthcare, many different personnel may be involved in a patient's care and continuity of that care is dependent on communication amongst staff providers. While it is possible that completing the VSCS may have primed respondents to consider these issues, the relative prevalence and potential severity of communication breakdowns warrant further investigation. In particular, the handoff from one provider to another can present a period of vulnerability (12), with risk of morbidity and mortality increasing in tandem with the number of handoffs (13). Students in the NCSU DVM program currently receive didactic training and laboratory instruction in team communication, as well as structured techniques for patient transfer. With this backdrop in mind, next steps should include expanding this training to reach technical staff and house officers, so they can then help introduce the cultural shift to faculty members. The adoption of standardized cognitive aids such as structured handoff forms and surgical safety checklists could help to formalize information transfer and improve patient safety outcomes (14). A surgical safety checklist has been developed, introduced in stages to the NCSU teaching hospitals, and is currently undergoing clinical piloting and revision.

The second factor contained statements focusing on the interpretation of managerial behaviors and response to error by staff. In general, survey responses were positive in this domain but a significant opportunity for improvement presents itself in the fact that only about 1/3 (27/77) of respondents indicated “I always feel able to question the decision or actions of someone with more authority.” Moreover, some of the narrative responses focused on concerns in this arena, e.g., “If an error occurs, there is still a lot of favoritism depending on who made the error” and “People are afraid to admit error due to the fear of punishment.” Most of these concerns were raised by staff members, in the context of their own fear about reporting an error. When a faculty member brought up the topic, it was presented as a reason why another person would be reluctant to report an error. Status asymmetry amongst team members leads to gaps in communication of critical safety information, and hierarchical issues resulting in the possibility of increased interpersonal conflict has been identified as a barrier to speaking up (15). Flattening of the traditional hierarchy can improve both communication and patient outcomes (16). Explicit interdisciplinary training in teamwork, communication, and human factor psychology has been shown to improve participants' confidence in speaking up when they perceive a patient safety risk (17).

Five statements co-localized within the third domain and all involved error and its management. Although most respondents agreed that procedures exist to trap errors and that positive changes have occurred as a result of errors, survey results indicated that formalized investigation and discussion of error does not regularly occur. A representative narrative statement about this issue includes: “At my former university employer, we had monthly routine ‘continuous quality improvement’ meetings where.errors were anonymously discussed and all employees were encouraged to offer ideas on how to prevent the errors in the future.” Formalized discourse surrounding error and negative patient outcomes (i.e., morbidity and mortality rounds) is a requirement in human healthcare settings that train residents (18). These discussions offer an enormous opportunity for learning as long as the focus is on process and systems improvement rather than “shame and blame” activity. Though individual review of a case by the Hospital Director and Service Chief could be initiated via request, formalized sessions discussing error and the systems that trap error were not widely in place at the time of survey administration. Since that time, a formalized Quality of Care Conference has been initiated within the Small Animal Teaching Hospital; however, the opportunity to formalize such discussions in the remaining clinical teaching areas exists.

The fourth domain identified within the NCSU VSCS contained three statements, which were co-localized in the Risk Perceptions domain of the NVSCS (8). Survey participants recognized the effect of stress on their own personal performance, as evidenced by the strong positive response to the statements in this factor.

Amongst items that did not load with any domain, of particular interest was the overwhelmingly negative response to the item that “The level of staffing is always sufficient to handle the number of patients.” Tellingly, one narrative statement indicated that an “…excessive workload and chronic understaffing lead to exhaustion and workplace errors.” Concerns about burnout, exhaustion, and staff shortages were primarily raised by staff members. In addition to demonstrating an understanding of the implications of system factors on patient safety, this further illustrates the share of physical and emotional burdens borne by support staff, as well as the perceived lack of respect and/or control over their working environment. The nature of these concerns illustrates the need to ensure that all members of the clinical team have a voice in establishing institutional safety culture and provides support for the implementation of efforts to support a flat hierarchy. A positive safety culture is one in which policies and procedures are developed to reduce and trap error before patient outcomes are impacted, including structural commitments such as appropriate staffing. An administrative commitment to identifying and rectifying understaffed clinical areas is an important opportunity to improve patient outcomes (19). A nationwide shortage of credentialed veterinary technicians exists, with resulting difficulties in finding qualified personnel to fill positions being noted as a major complaint by veterinary practices (20). The Bureau of Labor Statistics indicates that demand for veterinary technicians will grow 16.3% by 2029, contributing to an ongoing industry-wide shortage of veterinary technicians (21).

Two statements revealed underlying differences in perspective amongst respondents as stratified by professional role (Figures 1, 2): “Technician input is well-received in this practice” and “People who work here treat each other with respect,” with faculty members responding more positively than technicians and house officers. The difference in perspective may be reflective of wider issues within the veterinary professional community. Technicians report high levels of burnout with significant contribution from experiencing a low professional efficacy (22). Meaningful work requires the opportunity to utilize professional skills and knowledge as an effective part of the team (23). The fact that non-faculty members indicate that input from nursing staff is not always acknowledged appropriately indicates that significant improvements in job satisfaction and performance could be achieved by fostering a more collaborative workplace that recognizes the professional value of all individuals. In addition, the difference in perception of interpersonal interactions is concerning since disrespectful interpersonal interactions, including dismissiveness toward and humiliation of coworkers, can lead to inhibition of teamwork, avoidance behaviors that undermine communication, and decreased morale (24).

We have referred to the collection of attitudes regarding error, the handling of error, and systems to trap error as patient safety culture. However, there is some debate in the literature about the terms “safety culture” vs. “safety climate.” According to the developers of the SAQ, safety climate is probably a better term for the results of a survey as it refers to the collection of individual attitudes and perceptions at any one point in time whereas culture is a larger construct involving actual behaviors, policies, leadership roles, and overarching organizational values (5). However, there is much overlap in the literature and the two terms are often used interchangeably. We have followed the precedent of the NVSCS (8) and used the term patient safety culture.

Several limitations to this study exist, including relatively low response rates, especially among house officers. This could result in a self-selection bias in that people who felt strongly one way or the other may have been more likely to take the survey. Internet-based surveys typically suffer from low response rates (25), therefore, the Checklist for Reporting Results of Internet E-Surveys (CHERRIES) advises the calculation of view and participation rates rather than a response rate (26). Due to the anonymous treatment of participants by the web-based software, we were unable to collect this information.

Another limitation of the study is the inability to investigate patient safety culture at the clinical unit level. There are many different clinical groups within the three teaching hospitals at the NCSU CVM and it is likely that they harbor subcultures which would only be reflected in unit level surveys. In fact, several of the narrative statements focused on this issue: “More accurate information would be obtained by service specific questions,” “.there is a lot of variability among service centers.,” and “.the answers to these questions vary dramatically depending on the service,” However, due to the need to prevent indirect re-identification of participants in smaller service areas, we did not collect information about clinical unit of employment.

In conclusion, an adapted version of the NVSCS (8) demonstrated reasonable construct validity when administered to employees of a North American veterinary school. This is the first report of the deployment of such a survey at a veterinary teaching hospital in the United States. More investigation of this tool is warranted, including further psychometric testing of the instrument for our audience as well as in other clinical settings. Particularly interesting would be the comparison of survey responses to the original results after implementation of recommended structural patient safety improvements. In addition, comparison studies with other teaching institutions and deployment at the clinical unit level could be conducted.

## Data Availability Statement

The original contributions presented in the study are included in the article/supplementary material, further inquiries can be directed to the corresponding author/s.

## Ethics Statement

The studies involving human participants were reviewed and approved by Institutional Review Board, North Carolina State University. The patients/participants provided their written informed consent to participate in this study.

## Author Contributions

LL, SM, and RS-T conceived the study. M-WH assisted in modifying the original survey for distribution. LL and RS-T conducted the research and wrote the manuscript. JR conducted analysis and helped prepare the manuscript.

## Conflict of Interest

The authors declare that the research was conducted in the absence of any commercial or financial relationships that could be construed as a potential conflict of interest.

## Footnotes

^1^REDCap (Research Electronic Data Capture), REDCap Consortium.

^2^©The R Foundation, https://www.r-project.org/

^3^©The R Foundation, https://cran.r-project.org/web/packages/ltm/index.html

^4^©The R Foundation, https://cran.r-project.org/web/packages/lavaan/index.html

## Abbreviations

CVM: College of Veterinary Medicine
IRB: Institutional Review Board
NCSU: NC State University
NVSCS: Nottingham Veterinary Safety Culture Survey
SAQ: Safety Attitudes Questionnaire
VSCS: Veterinary Safety Culture Survey.
